# Pressurized Hot Water Extraction of Okra Seeds Reveals Antioxidant, Antidiabetic and Vasoprotective Activities

**DOI:** 10.3390/plants10081645

**Published:** 2021-08-10

**Authors:** Eng Shi Ong, Christina Liu Ying Oh, Joseph Choon Wee Tan, Su Yi Foo, Chen Huei Leo

**Affiliations:** Science, Math & Technology, Singapore University of Technology & Design, Singapore 487372, Singapore; engshi_ong@sutd.edu.sg (E.S.O.); christina_oh@sutd.edu.sg (C.L.Y.O.); joseph_tan@sutd.edu.sg (J.C.W.T.); suyi_foo@sutd.edu.sg (S.Y.F.)

**Keywords:** *Abelmoschus esculentus*, pressurized hot water extraction, principal component analysis, antioxidant, cytoprotective

## Abstract

*Abelmoschus esculentus L. Moench* (okra) is a commonly consumed vegetable that consists of the seeds and peel component which are rich in polyphenolic compounds. The aim of this study is to utilize pressurized hot water extraction (PHWE) for the extraction of bioactive phytochemicals from different parts of okra. A single step PHWE was performed at various temperatures (60 °C, 80 °C, 100 °C and 120 °C) to determine which extraction temperature exhibits the optimum phytochemical profile, antioxidant and antidiabetic activities. The optimum temperature for PHWE extraction was determined at 80 °C and the biological activities of the different parts of okra (Inner Skin, Outer Skin and Seeds) were characterized using antioxidant (DPPH and ABTS), α-glucosidase and vasoprotective assays. Using PHWE, the different parts of okra displayed distinct phytochemical profiles, which consist of primarily polyphenolic compounds. The okra Seeds were shown to have the most antioxidant capacity and antidiabetic effects compared to other okra parts, likely to be attributed to their higher levels of polyphenolic compounds. Similarly, okra Seeds also reduced vascular inflammation by downregulating TNFα-stimulated *VCAM-1* and *SELE* expression. Furthermore, metabolite profiling by LC/MS also provided evidence of the cytoprotective effect of okra Seeds in endothelial cells. Therefore, the use of PHWE may be an alternative approach for the environmentally friendly extraction and evaluation of plant extracts for functional food applications.

## 1. Introduction

*Abelmoschus esculentus L. Moench*, more commonly known as okra or lady’s finger is a flowering plant belonging to the Malvaceae family. It is an economical and important folklore medicine crop that was discovered in Ethiopia in the 12th century B.C. [1,2]. Each *A. esculentus* plant produces one pod, which has a light fuzz on the outer surface and contains many dark-coloured seeds in the interior of the plant [2]. Okra is a commonly consumed vegetable rich in carbohydrates, fibers, proteins and natural antioxidants [3,4,5]. It consists of the seeds and peel component, which are rich in polyphenolic compounds known to be the main bioactive components in the okra plant [3,4,5]. Specifically, polyphenolic compounds such as quercetin, isoquercetin, rutin, quercetin-3-O-gentibioside, hydroxycinnamic derivatives and catechin derivatives are present in okra [3,5]. Hence, okra extract may be developed into innovative products and alternative end-uses in the food and nutraceutical industries. The products may include functional foods with potent antioxidant and bioactive properties to promote health well-being [6,7].

Polyphenolic compounds such as flavonoids are well-regarded for their pleiotropic biological activities including antioxidant and vasoprotective actions [8]. Indeed, total polyphenolic extracts from okra Seeds is protective against oxidative stress-induced damage to rat liver cells [9]. Furthermore, okra extracts ingestion also reduces lipid peroxidation; augment the levels of catalase, glutathione peroxidase, and superoxide dismutase, and decreased glutathione in diabetes [10], which is a chronic disease manifested with increased oxidative stress-induced organ damage [11,12,13,14]. Generally, extraction yield and the biological activities of polyphenolic profile of the product is highly dependent on the extraction techniques, which have great effects on the utilization of polyphenolic compounds for functional food and pharmaceutical purposes [15,16]. Whilst there are several extraction techniques available for the extraction of functional food products, each extraction methods has its own advantages and limitations when the extraction efficiency, cost, convenience, time consumption and environmental impact is considered. Although the physiochemical characteristics, antioxidant activities and biological effects of okra have been reported, it is important to highlight that most of these studies used organic solvents (methanol or ethanol) to extract polyphenolic compounds from okra [4,6,7,9,17]. Extraction method that used more organic solvents will require proper waste disposal and solvent removal from the end product. In addition, toxic solvent such as benzene may arise from another solvent, for example, toluene or acetone where benzene is a known process impurity [18]. Therefore, utilization of solvent-free extraction technique will be a critical step that can greatly affect the end product without compromising on the biological effects of the functional food product [19,20].

Modern extraction techniques such as supercritical fluid extraction (SFE), pressurized hot water extraction (PHWE) and others have become more widely accepted for the solvent-less extraction of biologically active phytochemical compounds from various plant and food sources [19,20]. Despite the differences between using solvent and solvent-less extraction techniques, several earlier studies have shown that the chemical profile and biological activities of the botanicals extracted using these two methods are comparable. Taken together, this suggest that the use of solvent-less extraction methods may be an environmentally friendly and sustainable approach to extract and evaluatethe chemical compositions and biological activities of okra.

Given that different parts of okra may consist of distinct chemical composition and the biological activities, therefore, the objective of the current work is to utilize a solvent-free, PHWE method for the extraction of bioactive phytochemicals from different parts of okra. Specifically, a single step extraction will be carried out, the optimum temperature for PHWE extraction efficiency will be evaluated. Furthermore, different parts of okra will be characterized using antioxidant, antidiabetic assays and metabolite profiling of the vasoprotective effect of okra will be evaluated using LC/MS. Finally, determination of polyphenolic compounds, chemical fingerprint with pattern recognition tools such as principal component analysis (PCA) [21,22] will be used for the evaluation and comparison between different parts of okra extracts.

## 2. Materials and Methods

### 2.1. Chemicals and Okra Materials

Okra (mature and ready to be consumed) was purchased from local supermarkets in 2019 when the study is conducted. Azinobis-3-ethylbenzothiazoline-6-sulfonic acid (ABTS), 1,1-diphenyl-2-picrylhydrazyl (DPPH), ascorbic acid, Acarbose, HPLC grade water, sea sand and formic acid were purchased from Sigma-Aldrich (Singapore). All cell culture reagents, including trypsin, MCDB-131 media, Fetal Bovine Serum (FBS), Attachment Factor (AF), 0.4% trypan blue and L-glutamine acid were purchased from Thermofisher Scientific (Singapore). All other reagents used throughout the experiments in this study were of standard analytical grade.

The whole okra plants were washed and dried before they were separated into various parts, consisting of the Seeds, Outer Skin and Inner Skin. Subsequently, the various okra parts were oven-dried at 40 °C for 24 h [23]. The samples were crushed using mortar and pestle after being cooled down to generate a more homogeneous and representative sample. Samples were sieved individually to separate into following particle sizes: below 0.3 mm, between 0.3 mm and 2.8 mm, and above 2.8 mm. Fine samples with particle size below 0.3 mm was used for chemical analysis.

### 2.2. PHWE System and Heating under Reflux

For the extraction of active compounds from each sample, a laboratory PHWE system, which included a Shimadzu LC10 series pump (Kyoto, Japan), a stainless-steel extraction cell and temperature oven (Hewlett Packard 5890 Series II, Houston, TX, USA) was assembled and connected using stainless steel tubings as previously described [20]. Briefly, prior to loading the extraction cell, sample weight of 0.5 g was mixed with a small proportion of sand to maintain back pressure during the extraction process. A constant flow rate of 1.2 mL/min was set for the pump which recorded a pressure of 10–15 bar. Samples were extracted at different temperatures (60 °C, 80 °C, 100 °C and 120 °C) for 40–45 min into 50 mL of water. For heating under reflux, 2.5 g of sample and 100 mL of water was added. The extraction was allowed to continue for 3 h. Subsequently, the extracts obtained were dried down using a rotary evaporator concentrator plus (Eppendorf, Singapore) at 45 °C for approximately 1–1.5 h. The dried extracts were then weighed and reconstituted in deionized water to obtain stock concentrations for the extracts (Seeds: 16.28 mg/mL, Outer Skin: 11.45 mg/mL and Inner Skin: 10.45 mg/mL), which were used for subsequent experiments.

### 2.3. LC/UV/MS Profiling of Samples

Okra extracts was analysed using LC-UV-MS as described previously [23]. A C18 reverse phase HPLC column (Zorbax SB-C18- 3.5 microns, 10 cm × 4.6 mm) was used. Flow rate was at 0.25 mL/min and the analytical column was maintained at 40 °C. The injection volume was 5 µL. For LC/UV and LC/MS, the gradient elution consisted of mobile phase (A) 0.1% of formic acid in water and (B) 0.1% of formic acid in acetonitrile. For LC/UV and LC/MS, a gradient elution started at 0.10 min of 10% of B, increased to 100% B in 10 min and 5 min for column equilibration (total run time was 15 min). The electrospray Ionization (ESI) was in the negative mode, a nebulizing gas flow of 2.8 L/min, interface temperature of 300 °C with a heating and dry gas flow of 9 L/min. A UV detector was adjusted to detect polyphenolic compounds at the absorbance value of 280 nm. The derived extracts were filtered through a 0.2 µm filter into respective vials. The identified compounds (Table 1) in this study were based on various report and published MSMS spectra [3]. Previous studies noted that normalization to constant provided an alternative approach for the comparison of target compounds in complex matrix [24,25], hence calibration by external standard was not used.

For normalization to constant sum, the measured peak intensity was normalized to the total peak intensity for each sample derived from various parts of okra. For LC/UV, all peaks at 280 nm were obtained. For LC/MS, the peak intensities of their respective the molecular weight (*m*/*z*) were normalized to the total peak intensity of each sample to account for any differences in concentrations (Table 1). Subsequently, normalized data for each sample was condensed to 2 principal components using the PCA scores plot for 15 peaks. Observations on data clustering, trends and outliers were evaluated from the PCA scores plot.

### 2.4. Antioxidant Activity Assays

The antioxidant capacity of the okra extracts was evaluated based on the ability to scavenge free radicals using two different methods, DPPH and ABTS assays as previously described [23]. In the first set of experiments, the outer skin was extracted at different temperatures (60 °C, 80 °C, 100 °C and 120 °C) to determine the effect of extraction temperature on antioxidant capacity using DPPH assay. Briefly, the blank (200 µL of methanol), control (150 µL of water) and outer skin extracts (150 µL) from different temperature (60 °C, 80 °C, 100°C and 120 °C) were transferred into the 96-well plate. Consequently, excluding the blank, 50 µL of 100 µM DPPH solution were added into each well for incubation at 37 °C for 30 min. All experiments were performed in triplicates. In the subsequent set of experiments, the antioxidant capacities of the different okra parts (seeds, outer and inner) at 80 °C were compared. Similarly, the blank, control and various concentrations of the different okra parts (0.5 µg/mL to 5 mg/mL) were prepared into the 96-well plate. Subsequently, excluding the blank, DPPH solution (100 µM) was added into each well for incubation at 37 °C for 30 min. All experiments were performed in triplicates. Ascorbic acid (10 mM) was used as the positive control. The absorbance was recorded using a spectrophotometric plate reader (Thermofisher Multiskan GO) at 517 nm.

Absorbance change (%) = [(A_C_ − A_S_)/(A_C_ − A_P_) × 100], where A_C_ refers to the absorbance value of control, A_S_ refers to the absorbance measured from the extract sample and A_P_ refers to the absorbance measured from positive control.

For ABTS assay, 7 mM ABTS solution and 140 mM potassium persulfate solution were prepared. 88 µL of 140 mM potassium persulfate solution was added to 7 mM ABTS solution and the mixture was stored in the dark for 16 h. This produced a dark purple coloured solution, consisting of ABTS•+, which was diluted with distilled water (~50-fold) to give an initial absorbance of approximately 0.90–0.75 at 734 nm. Ascorbic standard curve (100 μM to 1 mM) was generated to produce 5–50% inhibition of the blank absorbance (ABTS•+ alone). Subsequently. 0.5 mg/mL of different okra parts were also prepared. 5 μL of each sample was added to 96-well microplate containing 200 μL of ABTS•+ solution. All experiments were performed in triplicates. The reaction was incubated for 30 min at room temperature, and absorbance was measured at 734 nm using Multiskan GO microplate reader (Thermo Scientific, Singapore). The absorbance at 734 nm was calculated as percentage of the absorbance of the uninhibited radical cation solution (Blank) according to the equation:

Absorbance change (%) at 734 nm = (1 − (A_S_/A_C_)) × 100, where A_C_ is the absorbance of uninhibited radical cation (ABTS•+ alone) and A_S_ is, the absorbance measured 30 min after the addition of antioxidant test samples.

The Vitamin C equivalent antioxidant capacity (CEAC) value of the okra samples was calculated using the equation obtained from the linear regression of the standard curve substituted of absorbance at 734 nm values for the okra sample.

### 2.5. Alpha-Glucosidase Assay

The α-glucosidase enzyme (0.5 U/mL, 50 µL) from *Saccharomyces cerevisiae* was first incubated with various concentrations of the different okra parts (2.5 µg/mL to 1.25 mg/mL, 50 µL) for 15 min at 37 °C in a 96-well plate. In order to account for the background absorbance readings induced by the higher concentrations of the okra extracts, sample blanks were prepared. In such cases, 50 µL of 100 mM of phosphate buffer (pH 6.8) was used to incubate with the various concentrations of the different okra parts (2.5 µg/mL to 1.25 mg/mL, 50 µL) under the same condition and duration. The antidiabetic drug, acarbose (100 mM) substituted the extracts as the positive control, while the negative control consisted of only the phosphate buffer and enzyme. Subsequently, 0.2 mM p-nitrophenyl glucopyranoside (pNPG) (100 µL) in phosphate buffer was added to initiate the reaction and the mixture was incubated at 37 °C for 15 min. After 15 min of incubation period, the α-glucosidase activity was recorded using a spectrophotometric plate reader (Thermofisher Multiskan GO, Singapore) at 405 nm. All experiments were performed in duplicates.

The α-glucosidase inhibitory activity was calculated using the formula: Absorbance change (%) = [100 − (A_E_ − A_EB_)/(A_C_ − A_CB_) × 100], where A_E_ refers to the absorbance value of extract, A_EB_ refers to the absorbance measured from the sample extract blank, A_C_ refers to the absorbance value of control and A_CB_ refers to the absorbance measured from control blank.

### 2.6. Cell-Culture and Cytoprotective Effects

Dermal microvascular endothelium (HMEC-1) cells were purchased from American Type Culture Collection (Manassas, VA, USA). HMEC-1 was cultured in MCDB-131, consisting of 2% FBS, 10–20 ng/mL of Recombinant Human Epidermal Endothelial Growth Factor (EGF), 10 µM of L-glutamic acid and maintained at the incubation temperature of 37 °C at 5% CO_2_. To evaluate the potential cytoprotective effects induced by the okra extracts, approximately 30,000 cells were seeded into the 6-well plate and left to adhere and proliferate for 24 h. Once the cells reached 80–90% confluency, the cells were challenged with H_2_O_2_ (0.1 mM) to cause cytotoxicity [23].

Simultaneously, different treatment (different parts of okra; Outer Skin, Inner Skin and Seeds extracts) was added to the cells at their respective IC_50_ values derived from the DPPH assay (OS: 0.29 mg/mL, IS: 1.46 mg/mL, Seeds: 0.014 mg/mL) The cells were incubated for 4 h at 37 °C and 5% CO_2_. To determine if okra extract treatment was able to prevent cell death, 2 mL of treatment medium (0.001 g/mL of filtered okra extract with 0.1 mM of H_2_O_2_ solution in MCDB-1 basal medium) was administered in duplicates. The cells were treated over a period of 4 h in 37 °C, 5% CO_2_ incubator. The cells were monitored and observed for its concentration and morphological alterations before determining cell viability by trypsinising and combining the duplicates of each extract for cell counting. The % cell viability was calculated using the formula [(C_a_/C_c_) × 100%], where C_a_ stands for no. of cells present in okra cells and C_c_ refers for the no. of cells present in the control set-up. A total of 6 replicates were performed independently for each different treatment. After the cell viability was determined, the treated cells were kept in a 1.5 mL micro-centrifuge tube at −20 °C for metabolites analysis using LC-MS.

Lipid extraction was performed in accordance to previously reported article with minor modifications [26,27]. The treated cells were removed from the freezer and left to thaw at r.t.p (room temperature and pressure) for 5 min. 1 mL of chloroform: methanol (3:1) was added to the cells and the samples were vortexed and centrifuged for 5 min at 10,000 rpm the supernatant was transferred into a new microcentrifuge tube and placed in a vapour centrifuge for 30 min at 45 °C. After which, 200 µL of methanol was added to the dried sample for LC-MS. The LC-MS analysis was used to identify standard metabolites to confirm the cytoprotective results obtained.

### 2.7. RNA Extraction and Quantitative Real Time PCR

To evaluate the potential anti-inflammatory effects exerted by the okra seeds, HMEC-1 were stimulated with the inflammatory cytokine, tumour necrosis factor-α (TNFα, 1 ng/mL) for 24 h. Our preliminary experiments demonstrated that TNFα treatment for 24 h induced vascular inflammatory through the upregulation of the expression of vascular adhesion molecules. Hence, for this assay, HMEC-1 were seeded into the 6-well plate and left to adhere and proliferate for 24 h. Once the cells were 80–90% confluent, HMEC-1 were treated with either TNFα (1 ng/mL) alone (control) or co-incubated with okra Seeds extracts (0.015 mg/mL and 0.05 mg/mL) for 24 h. After 24 h of treatment, the cells were washed with PBS and total RNA was extracted using the Bio-Rad AurumTM Total RNA Mini Kit (Cat.# 732-6820, Singapore) following manufacturers’ protocols. Quality and quantity of RNA was analysed using the NanoDrop^TM^ OneC Microvolume UV-Vis Spectrophotometer with A_260_:A_280_ ratios > 1.8 indicating sufficient quality for qPCR analysis. The RNA samples were reverse transcribed in a single run with a Bio-Rad T100^TM^ Thermal Cycler to produce complementary DNA (cDNA) using the Bio-Rad iScript cDNA Synthesis Kit, containing 0.5 µg of RNA in a final reaction volume of 20 µL.

The comparative cycle threshold (2-ΔΔCt) method of quantitative real-time polymerase chain reaction (qPCR) was performed as described previously [28,29]. Briefly, pre-customized human-specific forward/reverse primers were purchased from Sigma-Aldrich. Relative gene expression of vascular cell adhesion molecule 1 (*VCAM-1*), intercellular adhesion molecule 1 (*ICAM-1*), E-selectin (*SELE*) and C-C motif chemokine ligand 2 (*CCL-2*) were evaluated in HMEC-1 using Bio-Rad CFX96 Real-time PCR system (Biorad, Singapore). qPCR was performed using 96-well plates with 10 µL volume reactions in triplicate containing 10 µM of primers and SYBR Green master mix (Biorad, Singapore). Glyceraldehyde-3-Phosphate Dehydrogenase (GAPDH) was used as the reference gene. Negative template controls substituting cDNA with water or RT negative controls substituting the reverse transcriptase in the cDNA synthesis were included on each plate. For each sample, the mean GAPDH CT triplicate value was subtracted from the mean gene of interest triplicate CT value, and normalised to the reference gene (ΔCt). The data was normalised to their respective control (ΔΔCt) then presented as fold-change value (mean ± SD).

### 2.8. Statistical Analysis

Soft Independent Modelling by Class Analogy (SIMCA) software was used to generate the PCA plots. Group mean values were compared using one-way ANOVA to test for statistically significant differences between different extracted temperatures of okra as well as the different parts of okra with post-hoc analysis using Tukey’s test. The inhibitory concentration (IC_50_) value refers to the concentration of the okra extracts that exerts 50% of inhibitory effect. Concentration-response curves for DPPH and α-glucosidase inhibition were computer fitted to a sigmoidal curve using nonlinear regression (Prism version 5.0, GraphPad Software, San Diego, CA, USA) to calculate the sensitivity of DPPH and α-glucosidase inhibition for okra extract (IC_50_) [30]. Post-hoc analysis was only performed when the F value was significant and there was no variance in homogeneity [31]. All data are presented as mean ± SD. *p* < 0.05 was considered statistically significant.

## 3. Results and Discussion

### 3.1. Optimization of PHWE for Okra

**Figure 1:**
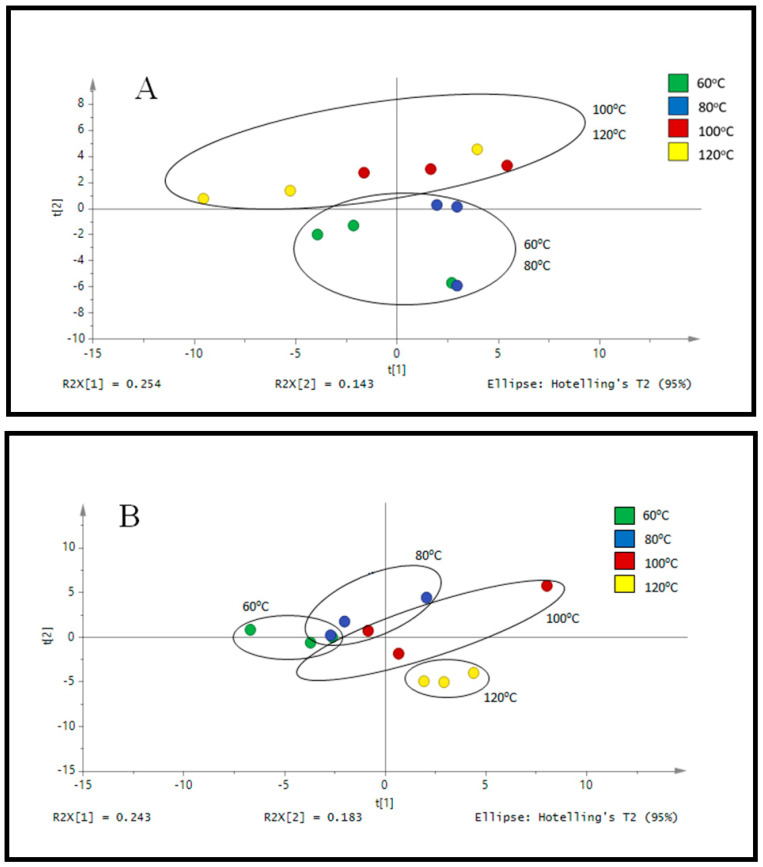
PCA score plot of (**A**) LC-MS profile of outer skin of okra and (**B**) LC-UV profile of outer skin at 254 nm.

For the optimization studies, the target compounds from the outer skin of okra were extracted with PHWE at 60 °C, 80 °C, 100 °C and 120 °C respectively. The compounds in the outer skin were determined by LC/UV and LC/MS respectively (Figure 1, Supplementary Table S1). Identification was based on the polyphenolic compounds reported in earlier work [3]. Different extraction temperature yielded different normalised peak area (%) for the selected polyphenolic compounds. The current approach of using normalization to constant sum was consistent with our earlier work [23]. At a higher temperature of 120 °C, the presence of polyphenolic compounds was significantly lower as compared to the rest, hence it was proposed that a higher applied temperature may cause polyphenolic compounds to be degraded [20]. In our earlier works, various marker or polyphenolic compounds in the botanical extracts were used to optimize the temperature of extraction [19,20]. We have observed that it was a challenge to select the optimum temperature of extraction as opposing trends for the selected compounds were noted.

From the chemical fingerprint obtained with LC/MS, a targeted approach based on selected ions was used (Supplementary Figure S2). From Supplementary Figure S2, based on the normalized peak area, the optimum temperature of extraction was reported at 80 °C to 100 °C, where the detected polyphenolic compounds seem to higher, although not statistically significant to the other extraction temperatures. From the PCA score plot in Figure 1A, it showed that distinctive profiles were obtained from okra samples extracted at 100 and 120 °C as compared to 60 and 80 °C. However, it remains inconclusive to establish that both temperatures produced similar phytochemical compound given that the distance between the points still have a certain gap. It was more evident that samples extracted at 60 to 80 °C have a completely distinct fingerprint as compared to 100 and 120 °C. Furthermore, the PCA score plot from the chromatograms obtained from LC/UV (Figure 1B) illustrated a high degree of similarity between the characteristic profile of okra samples extracted at 100 and 120 °C while extracts at 60 to 80 °C were quite distinct. Based on the data obtained, it was suggested that the variation in the profile of these are most likely to be attributed to the different extraction conditions and instrumental parameters that produced varying amount of phytochemical content. In addition, the chemical profile obtained from heating under reflux was compared to PHWE at different temperature. From PCA score plot in Supplementary Figure S1, it was observed to have a similar profile as compared to PHWE at 60 °C and 80 °C. PHWE does not involve the use of organic solvent during the extraction process and closely mimics how okra is normally cooked and consumed, hence, the presence of residue solvents such as benzene as a result of extraction process is minimal [18]. Furthermore, an earlier report also demonstrated that there was no toxicity or death observed when mice are administrated with high dose (2000 mg/kg, p.o.) of methanolic seed extracts of okra [32]. Although we have not done any quantification of such harmful compounds per se, we hypothesized that the presence of any toxic harmful compounds in the concentration of okra extracts used or the duration of treatment in this study is negligible to cause any negative effects. Furthermore, based on the concentration of okra extracts used and duration of treatment in this study, we did not observed any cell death or signs of cellular stress indicated by changes in cellular morphology. Hence, the monitoring of various polyphenolic compounds, chromatographic fingerprint with pattern recognition tools and antioxidant assay provided a practical approach to establish the quality of okra extracts.

**Figure 2:**
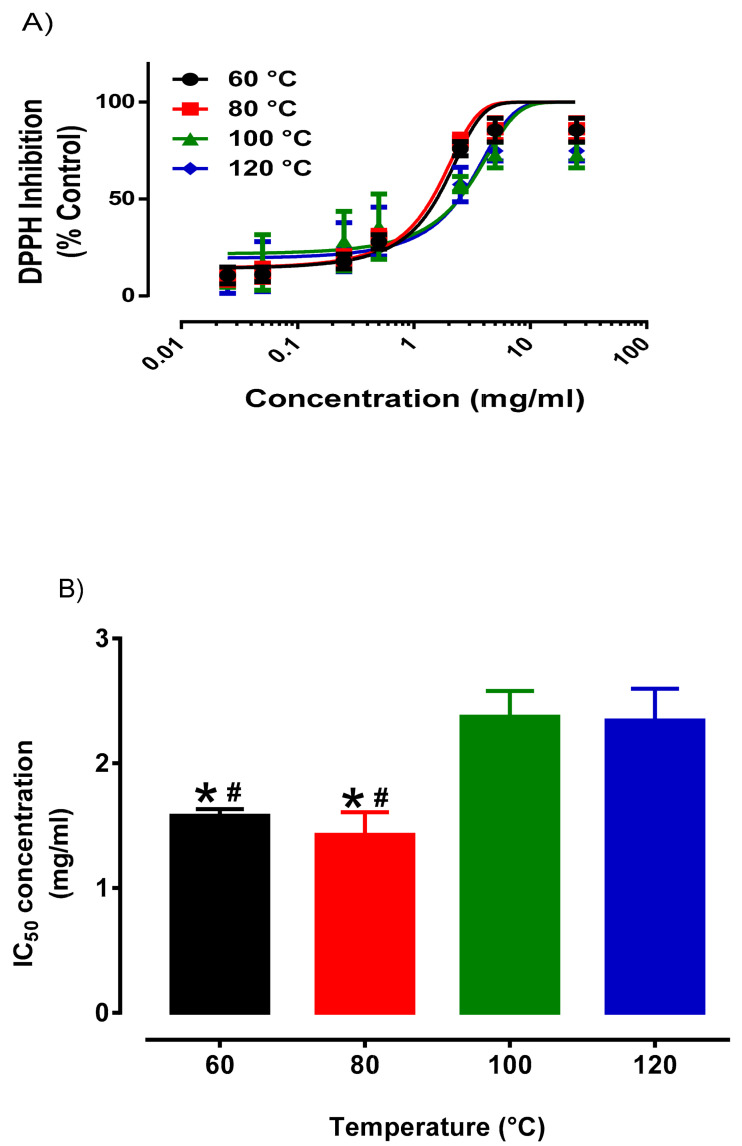
Antioxidant activity of outer skins of okra at different temperatures for PHWE (**A**): Concentration response curve of Outer Skin of okra against DPPH inhibition (**B**) Inhibitory Concentration (IC_50_) value derived from Figure 2A. * significantly different from 100 °C; ^#^ significant different from 120 °C. Data is presented as mean ± SD, *n* = 6.

It was noted that chemical fingerprint provided an approach for the quality assessment of the okra extracts obtained. Based on the monitoring of a single compound, it was a challenge to determine the optimum extraction temperature of okra. Hence, we assessed the antioxidant activity of the okra extracts at various extraction temperature to determine the optimum extraction temperature of okra. With regards to the comparison of various extraction temperature of okra extracts, concentration response curves of okra samples were evaluated for each temperature (Figure 2). From Figure 2, okra samples extracted at 60 °C (1.58 ± 0.053 mg/mL) and 80 °C (1.43 ± 0.07 mg/mL) has comparable IC_50_ values, whereas okra samples extracted at 100 °C (2.37 ± 0.21 mg/mL) and 120 °C (2.34 ± 0.25 mg/mL) has similar IC_50_ values. Okra samples extracted at 80 °C was significantly (*p* < 0.05) lower compared to sample extracted at 120 °C, indicated that okra samples extracted at 80 °C has the most potent antioxidant activity. Similarly, our preliminary experiment also suggests that the antioxidant activity (DPPH assay) of the okra extracts by heating under reflux was comparable with that obtained by PHWE at 60 °C and 80 °C (Data not shown). Furthermore, the antioxidant properties of okra extracts was similar to the calibration range of ascorbic acid (5 to 30 µM) reported [33]. Therefore, based on the chemical fingerprint of okra by using PCA score plots as well as the antioxidant activity (IC_50_ values), it was proposed that okra samples extracted 80 °C was the optimal temperature for PHWE.

### 3.2. Analysis of Different Parts of Okra by LC/UV/MS

**Figure 3:**
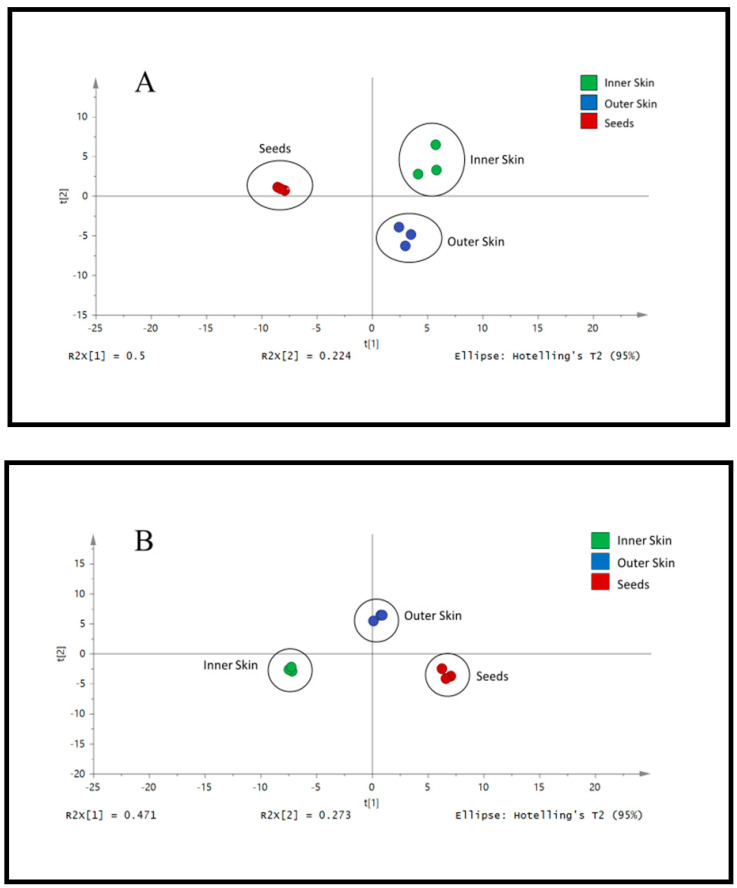
PCA score plot of (**A**) LC-MS profile of different parts of okra and (**B**) LC-UV profile of different parts of okra detected at UV 254 nm.

After establishing the optimum temperature of extraction for the selected phytochemical compounds present in the Outer Skin of okra, extraction of other parts of okra samples (Seeds and Inner Skin) was evaluated at 80 °C. From the PCA score plots in Figure 3, the blue points (Outer Skin), green points (Inner Skin) and red points (Seeds) were independently scattered. The data suggested that the phenolic profile for each part of okra were likely to be different (Figure 3, Supplementary Figure S3). Based on the chromatograms and TIC obtained, it was suggested the Seeds were found to contain a more varied content of phytochemicals (Supplementary Figures S4 and S5). Several polyphenolic compounds are measured in the okra, but differences were observed between various parts of okra (Table 1). Specifically, some of the compounds identified included p-coumaroyl-hexose, Quercetin 3-*O*-(malonyl)-glucose, quercetin-3-*O*-glucose-xylose, Sinapoyl-hexose, Sinapoyl-feruloyl and Kaempferol-3-*O*-glucose. Among all the compounds identified, Quercetin derivatives (Quercetin 3-*O*-(malonyl)-glucose & quercetin-3-o-glucose-xylose) were found to be the most abundant in okra. The okra seed sample was found to contain 6.79 ± 0.30% and 7.58 ± 0.39% of Quercetin-3-o-glucose-xylose and Quercetin 3-*O*-(malonyl)-glucose respectively. The outer skin of okra was found to have the highest content in Sinapoyl-feruloyl (3.50 ± 0.24%) as compared to inner skin (2.89 ± 0.04%) and seeds (0.084 ± 0.0048%). Inner skin of okra was found to contain the highest concentration of p-coumaroyl-hexose and Sinapoyl-hexose as compared to the outer skin and seeds (0.16 ± 0.018%). Okra Seeds had the highest concentration of Quercetin 3-*O*-(malonyl)-glucose, quercetin-3-o-glucose-xylose and Kaempferol-3-*O*-glucose.

Natural products can be divided into two major classes, such as primary and secondary metabolites. Primary metabolites are molecules essential for life, such as proteins, carbohydrates, fats and nucleic acid. In contrast, secondary metabolites are of limited occurrence and are often unique to the particular botanical. Plant secondary metabolites are considered as important sources of pharmaceuticals, food additives, flavours, cosmetics and others. For example. secondary metabolites in aromatic plants had been widely used for their preservative and medicinal values [34,35]. Flavonoids are one of the largest classes of natural phytochemical compounds that are known to have several biological functions conferring stress defense to plants and health benefits [8,36]. The isoflavone-aglycones, total phenolics, and biological properties (digestive enzyme inhibition; antioxidant) from leaves, leafstalks, roots, stems, seeds, and pods were observed to be different at different growth times of soybean plant [37]. With far-infrared radiation and hot air drying of pigmented rice, this is able to cause changes to either primary metabolites (amino acids) or secondary metabolites (polyphenolic compounds) [38]. From the PCA score plot in Figure 3, it was noted that a shift in the biosynthesis of secondary metabolites in the Outer Skin, Inner Skin and Seeds generated different amount of polyphenolic compounds. The distinctive chemical fingerprint of outer skin containing a higher amount of sinapoyl-feruloyl and other polyphenolic compounds may be needed to participate in the resistance response of plants to biotic and abiotic stresses. In addition, the shift in the secondary metabolite profile and the presence of quercetin 3-*O*-(malonyl)-glucose, quercetin-3-*O*-glucose-xylose, kaempferol-3-*O*-glucose and other polyphenolic compounds were also observed in the okra Seeds.

**Figure 4:**
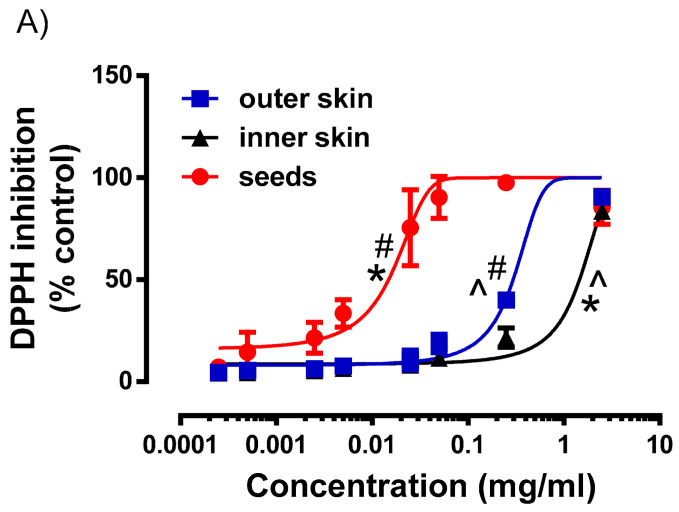

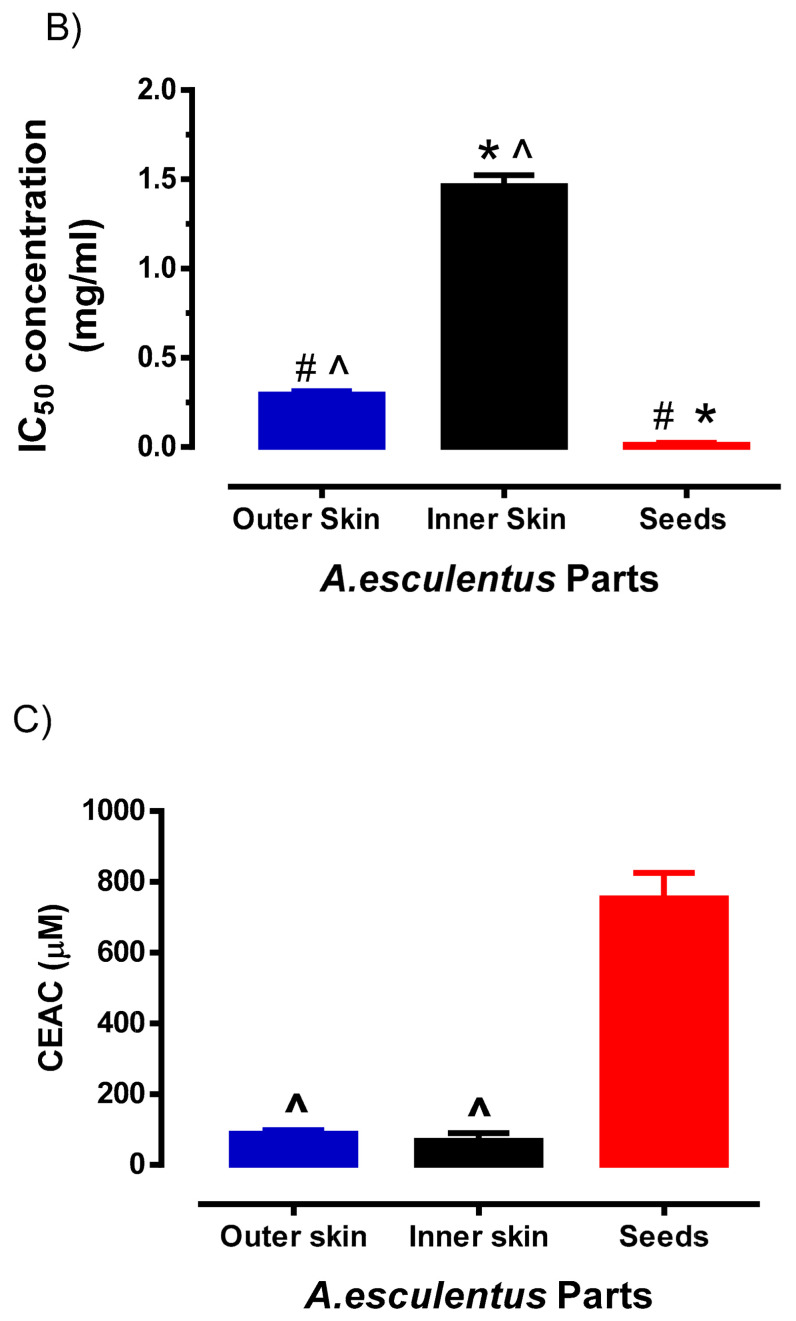
Antioxidant activity of different parts of okra with PHWE at 80 °C (**A**): Concentration response curve of various okra parts against DPPH inhibition (**B**) Inhibitory Concentration (IC_50_) value derived from Figure 4A. (**C**) Vitamin C equivalent antioxidant capacity (CEAC) of various okra parts. Data is presented as mean ± SD, *n* = 4–6. * significantly different from OS; ^#^ significant different from IS; ^ Significantly different from seeds.

While the different parts of okra contain various level of polyphenolic compounds, it remains unclear if their antioxidant capacity are affected. Hence, we evaluated the antioxidant capacity of the different parts of okra using two different antioxidant assays (Figure 4). The concentration response curve to DPPH inhibition by the different parts of okra were shown in Figure 4A. It was evident that the concentration response curve of the okra Seeds was the left most, indicating that okra seed extract was the most potent. The concentration response curve of the Outer Skin and Inner Skin were significantly shifted to the right in comparison to the okra Seeds (Figure 4A). Similarly, the okra Seed extracts demonstrated exceptionally low IC_50_ value (0.014 ± 0.01 mg/mL), which was significantly (*p* < 0.01) lower when compared to Outer Skin (0.29 ± 0.06 mg/mL) and Inner Skin (1.46 ± 0.004 mg/mL) okra extracts respectively (Figure 4B). To further validate the antioxidant effects of the various okra part extracts, a secondary ABTS antioxidant capacity assay was performed. In this experiment, the CEAC of the Seeds was significantly higher (*p* < 0.05, 1-way ANOVA, Tukey’s test) than the Inner and Outer Skin of okra. Consistent with the DPPH antioxidant assay, the CEAC of the Seeds was about 10-fold more potent than the Inner and Outer Skin of okra. In this study, the antioxidant activities of various parts of okra as determined by DPPH and ABTS assay was consistent with other reports based on the polyphenolic profiles of okra fruits at various stages [3,7] as well as the methanolic seed extract [32]. Moreover, the antioxidant activities of the extracts from PHWE were consistent with what was obtained from polysaccharides extracted from okra [39].

**Figure 5:**
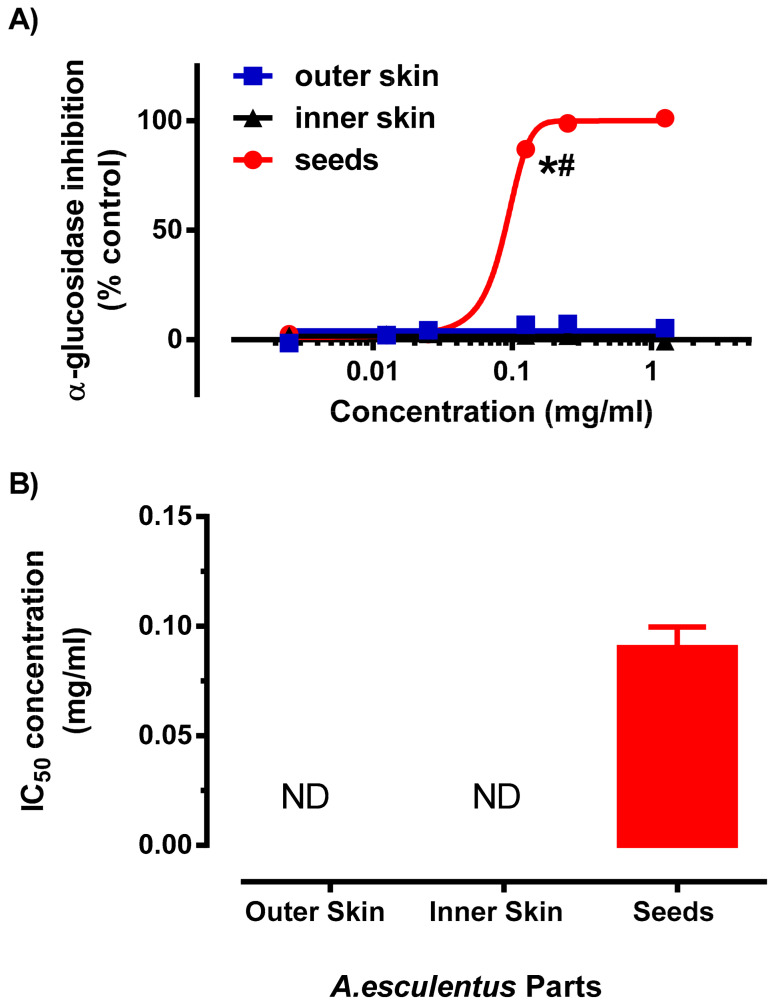
Inhibition of α-glucosidase activity of different parts of okra with PHWE at 80 °C (**A**): Concentration response curve of various okra parts against α-glucosidase inhibition (**B**) Inhibitory Concentration (IC_50_) value derived from Figure 4A. Data is presented as mean ± SD, *n* = 3–4. * significantly different from OS; ^#^ significant different from IS; ND: not determined.

In addition to antioxidant activity, we also evaluated the antidiabetic capacity of the different parts of okra using α-glucosidase inhibition assay (Figure 5). It was evident that the concentration response curve of the okra seed was a sigmodal curve, reaching maximal (~100%) inhibition of the enzyme at the highest concentration which was significantly (*p* < 0.01) higher when compared to Outer Skin and Inner Skin okra extracts. The maximum efficacy of the okra Seeds was comparable to the positive control, antidiabetic drug, acarbose (100 mM), which produced 95.5 ± 0.4% (*n* = 4) inhibition (Figure 4A). Similarly, the okra Seed extracts demonstrated exceptionally low IC_50_ value (0.01 ± 0.01 mg/mL) for α-glucosidase activity (Figure 4B). Interestingly, the concentration response curve of the Outer Skin and Inner Skin were flat, indicating that the Outer Skin and Inner Skin do not have any inhibitory effect on the α-glucosidase (Figure 4A,B). Consistent with previous reports, okra extracts also exhibited inhibitory response towards α-glucosidase and treatment of okra subfractions reduced blood glucose levels in diabetic animal models [40,41]. However, our results suggest that the antidiabetic effect of okra, at least through the inhibition of α-glucosidase, was largely mediated by the okra Seeds with little to no effect contributed by the Outer Skin and Inner Skin. Different cultivars of okra were demonstrated to yielded distinctive α-glucosidase inhibition. Although the cultivar of okra was unknown in this study, the IC_50_ value for α-glucosidase activity found in this study was comparable to previously reported study [42].

Therefore, both in vitro antioxidant and α-glucosidase assay suggest that the okra Seeds have higher antioxidant and antidiabetic capacity compared to the Inner and Outer Skin of okra, which is likely to be underpinned by distinct composition of polyphenolic compounds.

### 3.3. Cytoprotective Effects of Okra Extracts in Endothelial Cells

**Figure 6:**
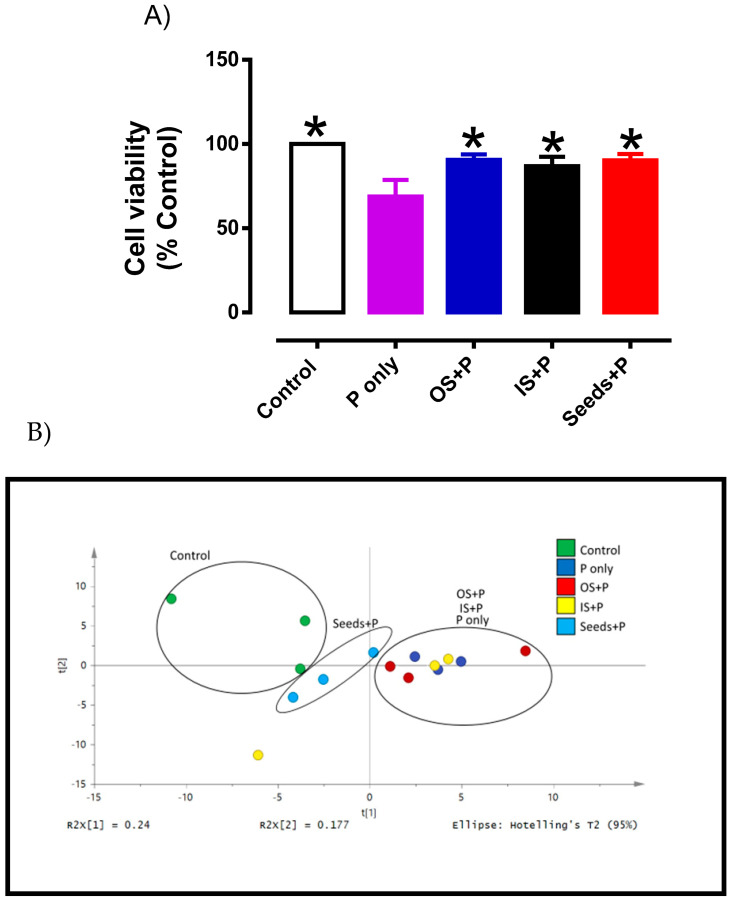
(**A**) Cytoprotective effects of okra parts on HMEC-1 cells. HMEC-1 cells were treated with 100 µM H_2_O_2_ only (P only) or co-treated with various okra parts (OS, outer skin; IS, inner skin and Seeds). Data is presented as mean ± SD, *n* = 6. * significantly different from P only (**B**) PCA score plot of LC-MS metabolites profile of control and treated HMEC-1 cells with different parts of okra (*n* = 3).

Phytochemicals are also known as non-essential nutrients with diverse health benefits such as antioxidant capacity [6,7]. In this study, it was noted that okra Seeds consist of several phytochemicals which is accompained by antioxidant capacity using the DPPH assay. The antioxidant capacity of okra Seeds extracted from PHWE was consistent with earlier reports, even though other extraction methods were used [6,7]. Though the DPPH/ABTS method is a convenient assay to determine antioxidant activity, it may not be a true representation of cellular antioxidant capacity. Therefore, the cytoprotective effect of the various okra extracts were investigated in endothelial cells (HMEC-1). Endothelial dysfunction is a critical and initiating factor for the development of cardiovascular complications in several diseases, including diabetes, hypertension and reproductive disorders [43,44]. It is well-established that increased production of reactive oxygen species and inflammation are important contributors to endothelial dysfunction [45,46,47]. In this study, HMEC-1 cells were treated with H_2_O_2_ to induce cellular damage, which was evident by a significant decrease (*p* < 0.05) in cell viability when compared to control (Figure 6A). In order to test if the antioxidant activity of the okra extracts were able to prevent oxidative stress-induced cellular damage, HMEC-1 cells were co-treated with the various okra extracts. Using their respective IC_50_ concentrations, co-treatment of various okra extracts with H_2_O_2_ significantly increased cell viability in comparison to H_2_O_2_ treatment alone (Figure 6A). Taken together, the data proposed that different part of okra has the capacity to protect vascular cells from oxidative damage. Specifically, polyphenolic compounds in okra Seed extracts exhibited the strongest antioxidant activity among the okra Inner and Outer Skin as it requires the least concentration to produce similar cytoprotective effects.

Lipids are one group of small molecules that have many key biological functions. These include serving as structural components of cell membrane, energy storage and others [48]. Oxidative modifications of lipids and other biomolecules induced by reactive oxygen species has been implicated in the progress of many diseases [11,49,50,51,52]. The presence of oxidative stress causes changes in the lipid profile and antioxidants from natural sources may their protective roles either directly or indirectly in the physiological defense network to inhibit oxidative modification of lipids [53,54]. Therefore, to further characterize the potential vascular protective effects of okra extracts, metabolite profiling of several lipid markers was analysed in HMEC-1 cells (Supplementary Table S2). The PCA score plot was used based on the endothelial cells to measure metabolite concentrations between the control, H_2_O_2_ treatment alone and H_2_O_2_ co-treated with okra extracts (Figure 6B). A clear separation in metabolic profile was observed between the control and H_2_O_2_ treatment, indicating that a marked alteration of metabolites in the endothelial cells (Figure 6B). Interestingly, despite exhibiting antioxidant activity, only okra Seeds co-treated with H_2_O_2_ shifted the metabolic profile closer to the control, whereas okra Outer and Inner Skin co-treatment displayed similar metabolic profile that were closer to H_2_O_2_ treatment. Although no statistical differences was observed at a specific metabolites level (Supplementary Table S2), based on the PCA score plot our preliminary data suggested that okra Seeds treatment appears to restore the whole metabolomics profile (known and unknown metabolites) of H_2_O_2_-induced oxidative damage to comparable levels as control. Furthermore, okra Inner and Outer Skins appeared to be less active in their ability to reverse metabolic profile of the lipid markers.

**Figure 7:**
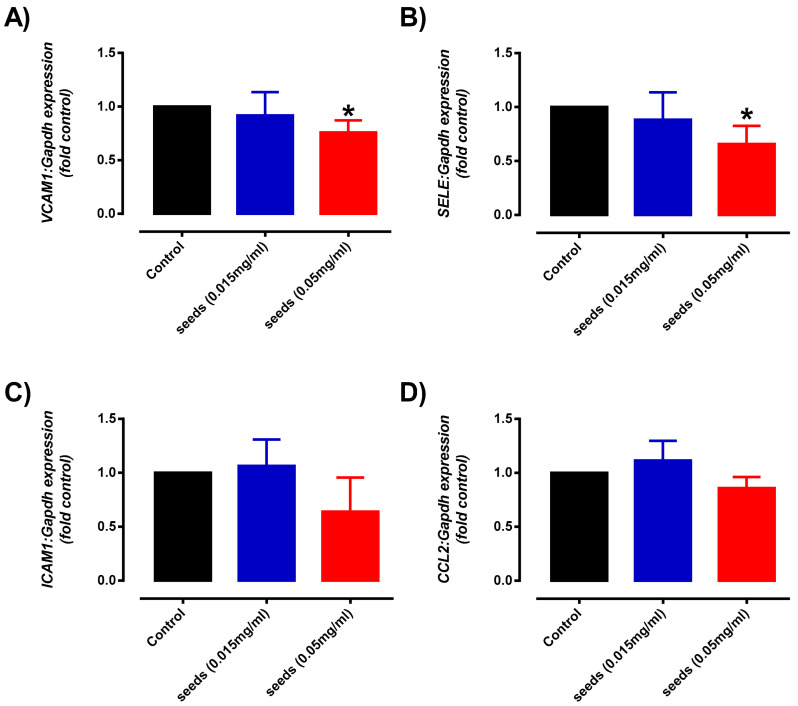
Quantitative analysis of (**A**) *VCAM1*, (**B**) *SELE*, (**C**) *ICAM1* and (**D**) *CCL2* mRNA expression in Tumour-necrosis factor-α (TNFα, 1 ng/mL) exposed HMEC-1 cells treated with either control or okra Seeds (0.015 and 0.05 mg/mL) for 24 h. Data is presented as mean of 2^−ΔΔCt^ ± SD, *n* = 4–6. * Significantly different to control, *p* < 0.05, (1-way ANOVA, Dunnetts’s post-hoc test).

Given that okra Seeds have the most effective antioxidant and cytoprotective activity, their potential anti-inflammatory effects were further investigated. In this study, a well-established model of TNFα-stimulated vascular inflammation was employed to evaluate the anti-inflammatory effects of okra Seeds. Consistent with numerous previous studies, our preliminary data also showed that TNFα treatment upregulated the expression of several adhesion molecules (*VCAM-1*, *ICAM-1* and *SELE*) and inflammatory cytokines (*CCL-2*) in endothelial cells, which are highly associated with vascular inflammation and endothelial dysfunction [46,55,56]. In the HMEC-1 co-treated with higher concentration but not lower concentration of okra Seeds extract, the gene expression of *VCAM-1* (Figure 7A) and *SELE* (Figure 7B) were significantly decreased after 24 h of treatment. Although the gene expression of *ICAM-1* (Figure 7C) showed similar downregulation trend, this effect induced by okra Seeds treatment did not reach statistical significance. There was no effect of okra Seeds treatment on *CCL-2* (Figure 7D) expression in TNFα-stimulated HMEC-1. Consistent with our data, a recent study also reported that chronic treatment with whole okra extract for 8 weeks reduced high-fat diet-induced inflammatory markers in the endothelial cells [57]. Taken together, our results suggest that okra Seeds appears to reduce endothelial inflammation, which is an important risk factor for the development of cardiovascular complications in several diseases, including diabetes and hypertension.

The current work revealed that the types of phytochemicals and characteristics of different parts of okra were found to contribute to the antioxidant and cytoprotective activity. In this study, various okra parts were extracted using PHWE and yielded similar chemicals compounds when compared to earlier studies where other extraction methods such as supercritical fluid extraction or ethanol extraction [3]. Despite having comparable chemical profile and biological activities, PHWE method is reported to be more cost effective in manufacturing and environmentally sustainable. Specifically, it was reported that the manufacturing cost of extracting target compounds from New Zealand grape marc using subcritical water extraction or other solventless extraction techniques (PHWE) was estimated to be NZ$87-89.60/kg. This was considerably much below in comparison to the cost of manufacturing using the supercritical fluid extraction (NZ$123.40/kg). In addition to cost effectiveness, solventless extraction also has less environmental impact in comparison to ethanol extraction. For instance, the potential environmental impact (PEI) of using ethanol was reported at 1.91 PEI/tonne, whereas there is zero net PEI by using water extraction [58]. Through life cycle assessment, the usage of ethanol extraction may have moderate gains on extraction yield, but increased the PEI [59]. Hence, the data in our current work is consistent with other reports [7,32,39,42,58,59], suggesting that PHWE is an environmentally friendly and likely to be a cost-effective technique for the extraction of natural products that are biologically active.

### 3.4. Conclusions

Green extraction method such as PHWE is a solvent free approach for the preparation of okra extracts. Chemical fingerprinting with PCA provided an alternative for the chemical standardization of plant extracts. The combination of chemical standardization such as LC/UV and LC/MS, pattern recognition tools and biological assays provided an approach for the evaluation of okra seed extracts for functional food. Finally, it was noted that solvent free extracts of okra seeds have antioxidant, antidiabetic capacity and vasoprotective action.

## Figures and Tables

**Table 1:** Chromatographic and mass spectrometric analysis of polyphenolic compounds identified in different parts (Outer Skin, Inner Skin and Seeds) of *A. esculentus* extracted at 80 °C PHWE. Values are represented as mean ± SD (*n* = 3). * significantly different from OS; ^#^ significantly different from IS; ^^^ Significantly different from Seeds. t_R_: Retention time, ND: Not detected.

Polyphenolic Compounds	t_R_ (min)	MS^-^ (*m*/*z*)	MS/MS (*m*/*z*)	Quantitative Analysis (Normalised % Peak Intensity)		
				Outer Skin (OS)	Inner Skin (IS)	Seeds
p-courmaryol-hexose	0.923	326	147, 164	0.31 ± 0.04 ^#^	0.84 ± 0.09 *^^^	0.16 ± 0.02 ^#^
Sinapoyl-feruloyl	2.86	399	193, 207	3.51 ± 0.24 ^#^^	2.89 ± 0.04 *^^^	0.084 ± 0.005 *^#^
Quercetin-3-O-glucose-xylose	3.421	595	300	5.08 ± 0.63 ^#^^	0.81 ± 0.15 *^^^	6.80 ± 0.30 *^#^
Quercetin 3-O-(malonyl)gluose	3.877	549	301	0.15 ± 0.21	0.52 ± 0.03 ^^^	7.57 ± 0.39 *^#^
Kaempferol 3-O-glucose	3.97	447	285	ND	ND	0.559 ± 0.001 *^#^
Sinapoyl-hexose	4.584	385	193, 176	0.36 ± 0.17	0.82 ± 0.28 ^^^	0.11 ± 0.01 ^#^

## Data Availability

The data presented in this study is available in the article and Supplementary Materials.

## Supplementary Materials

The following are available online at https://www.mdpi.com/article/10.3390/plants10081645/s1, Figure S1: PCA loading plot of (A) LC-MS profile of outer skin of okra and (B) LC-UV profile of outer skin at 254 nm; Figure S2: PCA score plot of LC-UV profile of outer skin of okra detected at UV 254 nm; Figure S3: PCA loading plot of (A) LC-MS profile of different parts of okra and (B) LC-UV profile of different parts of okra detected at UV 254 nm; Figure S4: Chromatograms of (A) Inner Skin extracts from okra, (B) Outer Skin extracts from okra and (C) Seeds extracts from okra; Figure S5: Total Ion Chromatograms (TIC) of (A) Inner Skin extracts from okra, (B) Outer Skin extracts from okra and (C) Seeds extracts from okra; Table S1: Chromatographic and mass spectrometic quantitative analysis of polyphenolic compound identified in A; Table S2: List of metabolites identified in control and treated HMEC-1 cells by different parts of Okra.
