# Supplementary material for: Pressurized Hot Water Extraction of Okra Seeds Reveals Antioxidant, Antidiabetic and Vasoprotective Activities
Source: Plants (Basel). 2021 Aug 10;10(8):1645. doi: 10.3390/plants10081645 (PMC8399463; doi:10.3390/plants10081645)
Supplement: Supplementary file 1 [file plants-10-01645-s001.zip › plants-1316856-supplementary.pdf]

Supplementary Information

Pressurized hot water extraction of okra seeds reveals antioxidant, antidiabetic and vasoprotective activities

Eng Shi ONG[1,*], Christina Liu Ying OH[1], Joseph Choon Wee TAN[1], Su Yi FOO[1], Chen Huei LEO[1,*]

[1]Science, Math & Technology, Singapore University of Technology & Design, Singapore 487372

***Co-corresponding Author:**

Dr Chen Huei LEO,

Singapore University of Technology and Design,

8 Somapah Road, Singapore 487372, Republic of Singapore

E-mail address: chenhuei_leo@sutd.edu.sg

Tel: +65 6434 8213

Dr Eng Shi ONG,

Singapore University of Technology and Design,

8 Somapah Road, Singapore 487372, Republic of Singapore

E-mail address: engshi_ong@sutd.edu.sg

Tel.: +65 6499 4513. Fax: +65 67795161.

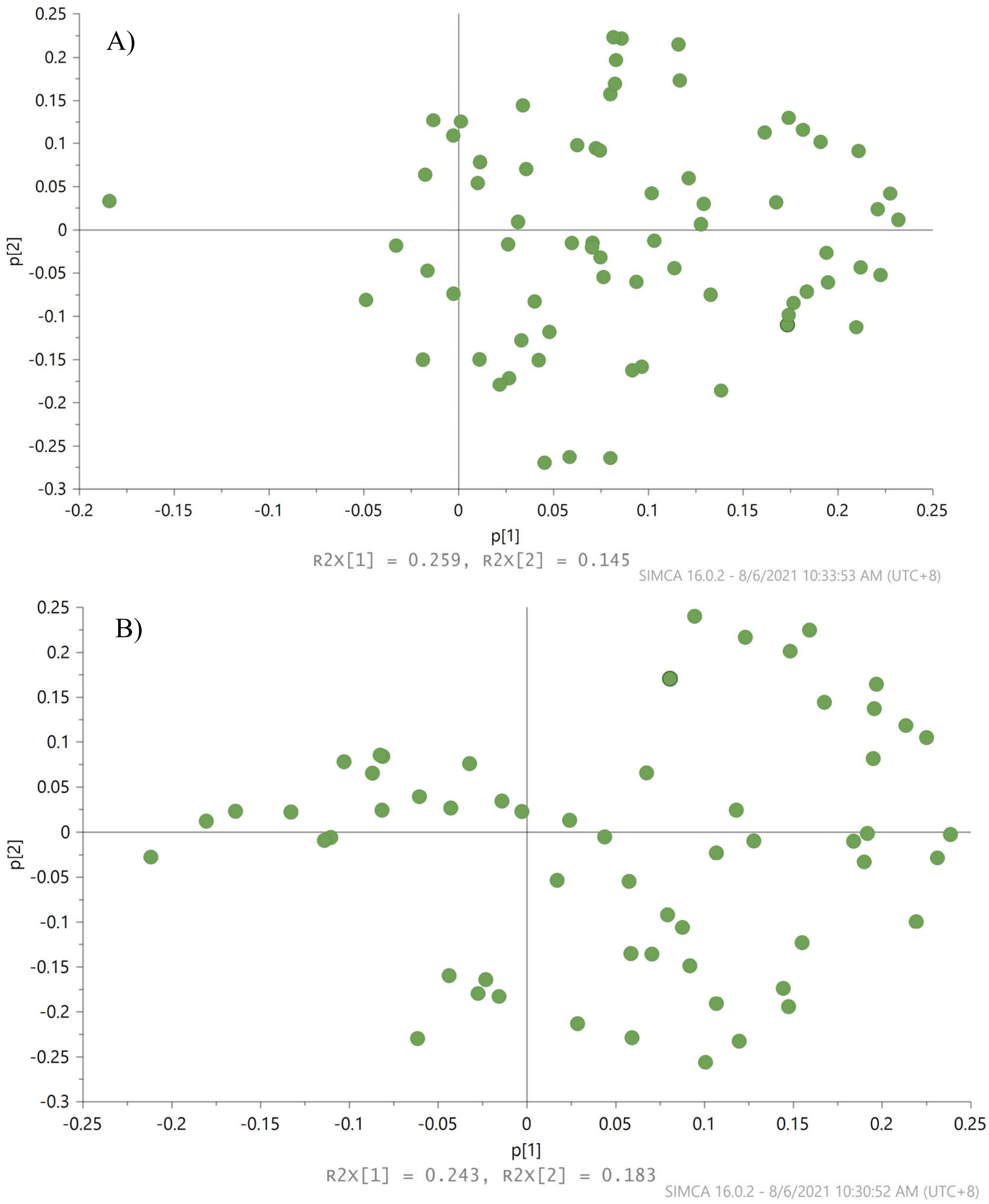

Figure S1. PCA loading plot of (A) LC-MS profile of outer skin of okra and (B) LC-UV profile of outer skin at 254 nm.

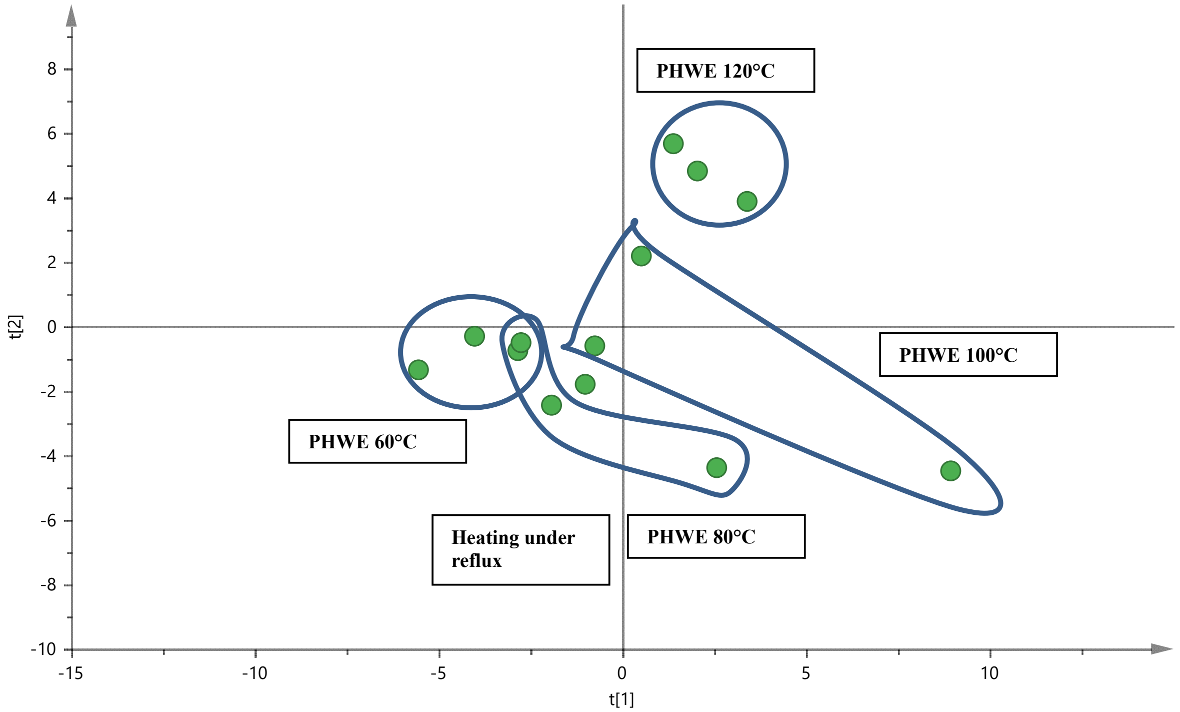

Figure S2: PCA score plot of LC-UV profile of outer skin of okra detected at UV 254 nm. Unmarked point was the data point generated by heating under reflux.

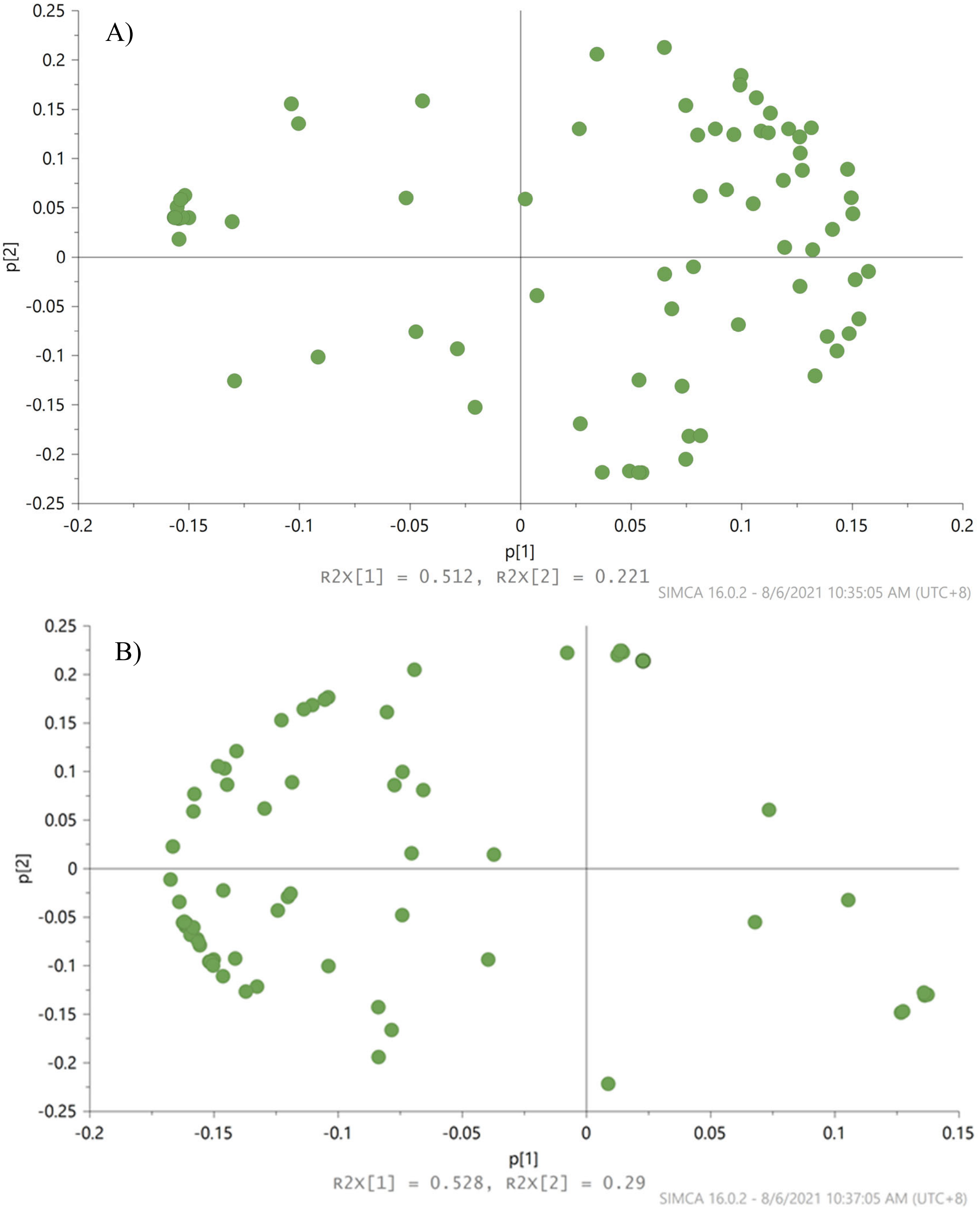

Figure S3: PCA loading plot of (A) LC-MS profile of different parts of okra and (B) LC-UV profile of different parts of okra detected at UV 254nm.

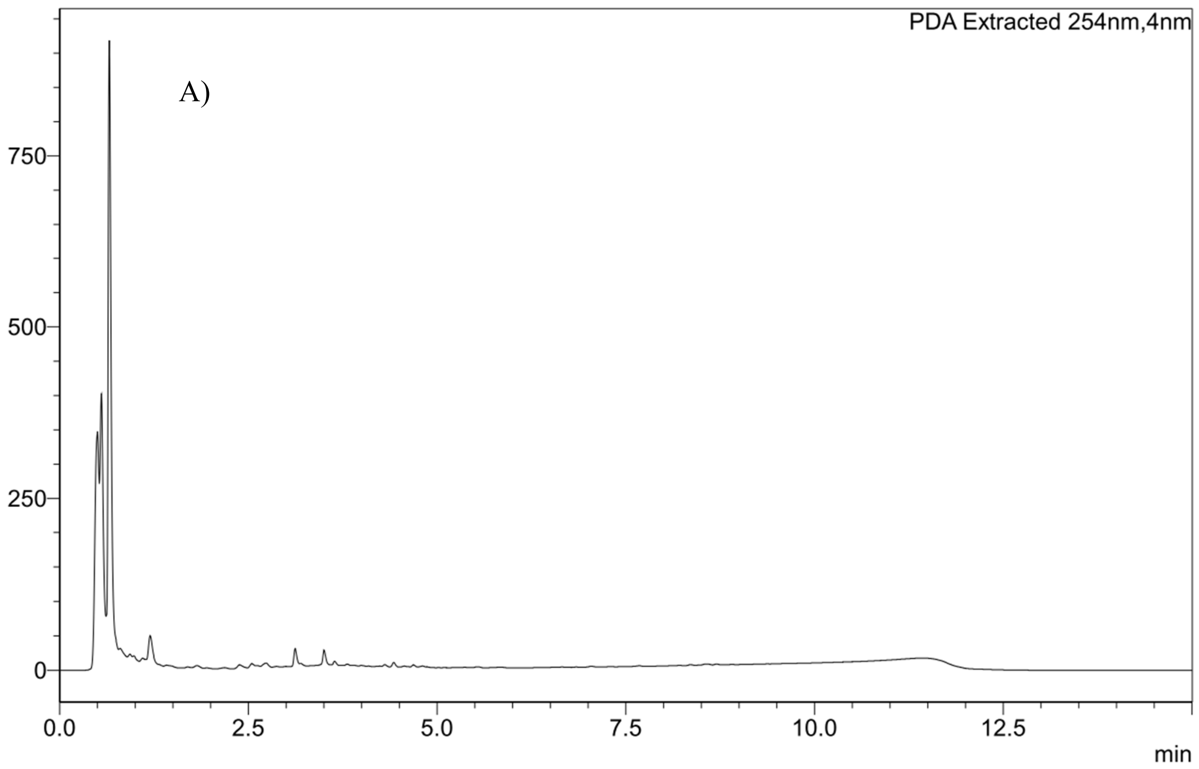
PDA Extracted 254nm,4nm
A)
750
500
250
0
0.0
2.5
5.0
7.5
10.0
12.5
min

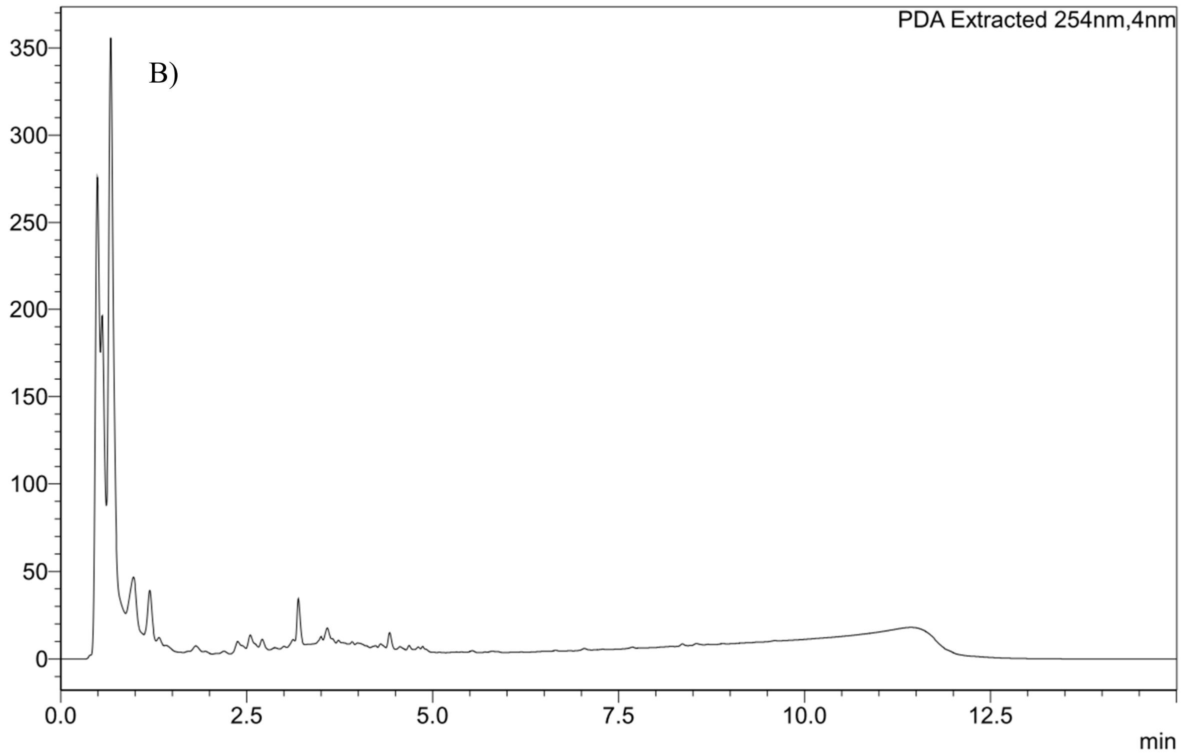
PDA Extracted 254nm,4nm
B)
350
300
250
200
150
100
50
0
0.0
2.5
5.0
7.5
10.0
12.5
min

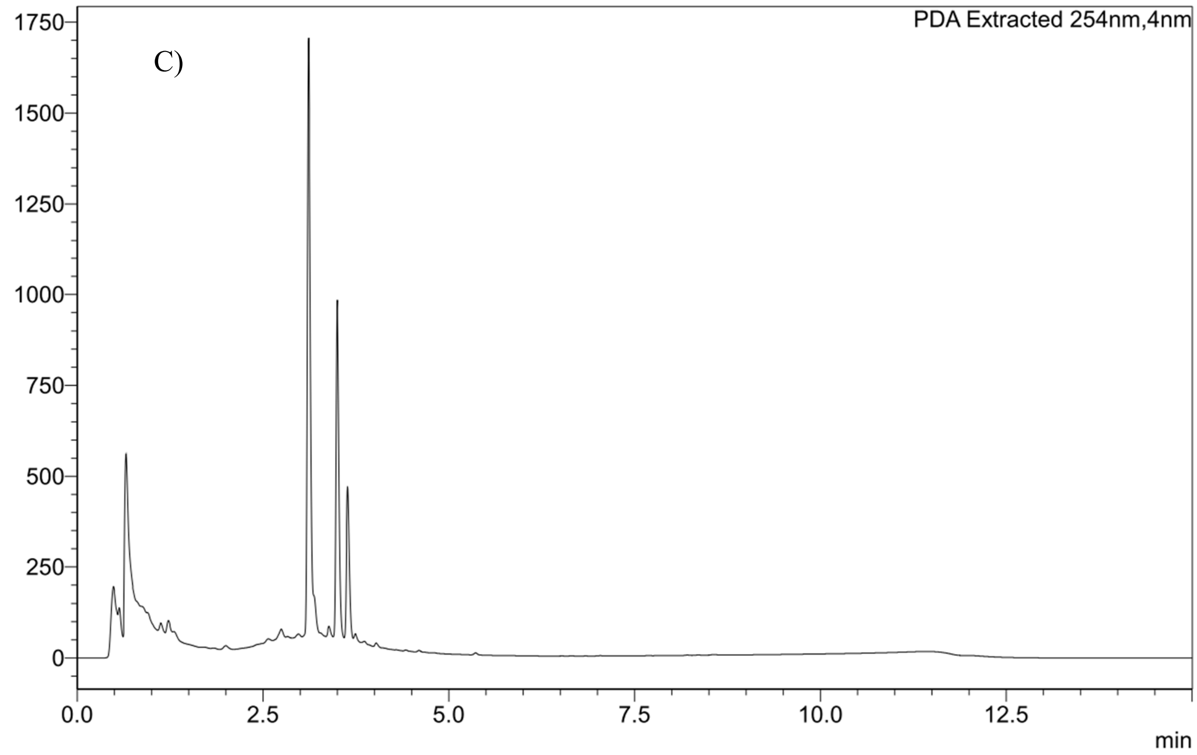

Figure S4: Chromatograms of A) Inner Skin extracts from okra, B) Outer Skin extracts from okra and C) Seeds extracts from okra.

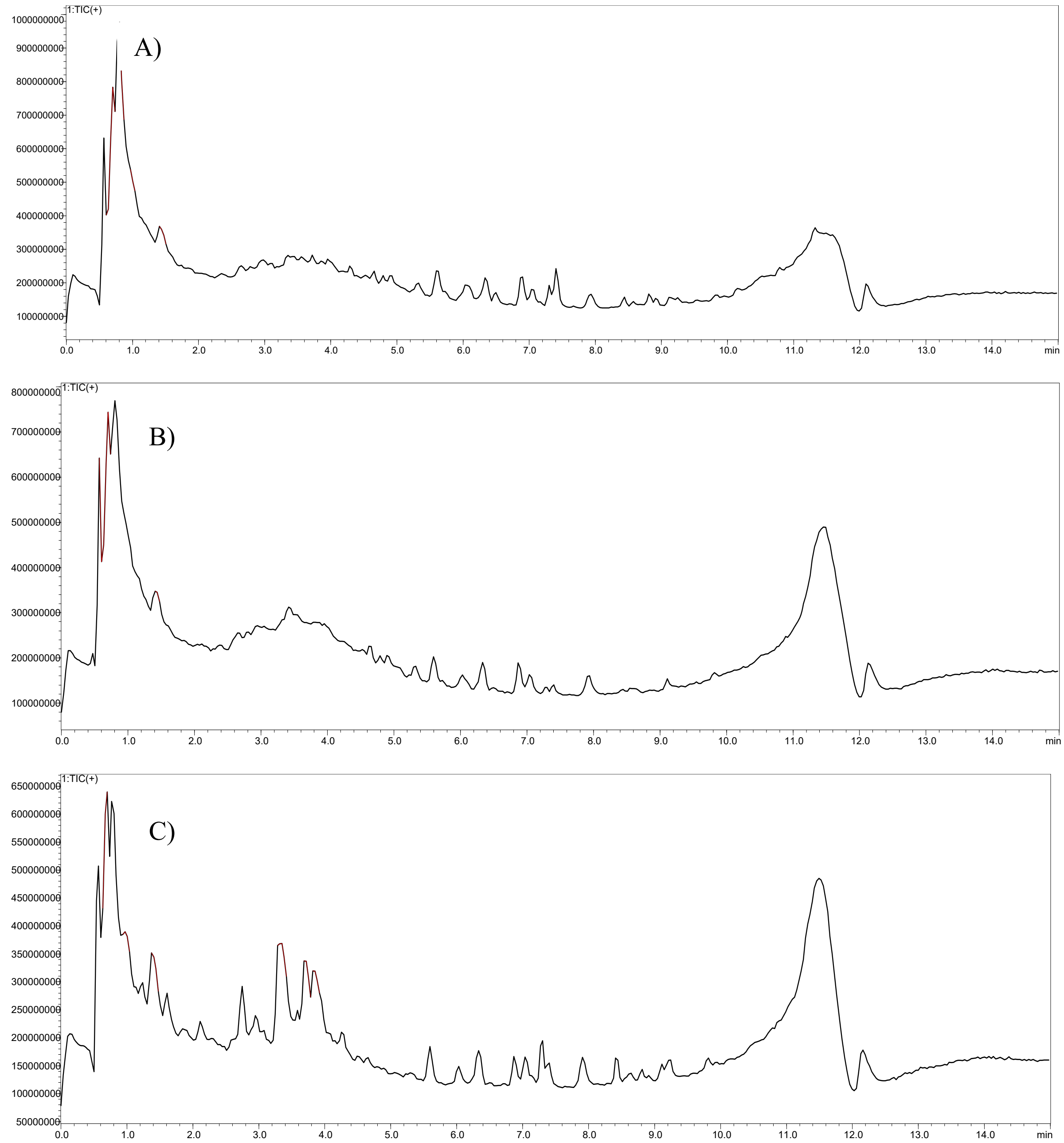

Figure S5: Total Ion Chromatograms (TIC) of A) Inner Skin extracts from okra, B) Outer Skin extracts from okra and C) Seeds extracts from okra.

Table S1: Chromatographic and mass spectrometic quantitative analysis of polyphenolic compound identified in A. esculentus subjected to PHWE at various temperature, 60°C, 80°C, 100°C and 120°C**.** Values are represented as mean ± SD (n=3). *significantly different from 120°C, $P<0.05$, One-way ANOVA, Tukey's test Abbrievations: t_R: Retention time

Polyphenolic Compound	t_R (min)	MS^- (m/z)	MS/MS (m/z)	Quantitative Analysis (Normalised % Peak Intensity)			
				60°C	80°C	100°C	120°C
p-courmaryol-hexose	0.863	326	147, 164	0.23±0.05	0.29±0.02*	0.18±0.08	0.11±0.06
Sinapoyl-ferloyl	2.413	399	193	3.14±0.92	4.23±0.09	4.41±0.72	2.8±2.1
Quercetin-3-O-diglucose	3.281	625	301	0.12±0.04	0.31±0.10	0.24±0.14	0.19±0.14
Quercetin-3-O-glucose-xylose	3.342	595	371, 300	2.21±0.98	3.65±0.48	3.77±1.05	2.45±1.73
Catechin dimer	3.632	579	285	0.24±0.09	0.16±0.04	0.14±0.04	0.19±0.08

Table S2. List of metabolites identified in control and treated HMEC-1 cells by different parts of Okra (IS: inner skin, OS: outer skin)

Values are represented as mean±SD (n=3), ND: not detected

Identified Metabolites	MS Mode	t_R (min)	MS (m/z)	Normalised Peak Intensity (%)				
				Control	Cells treated with peroxide	Cells treated with peroxide and OS	Cells treated with peroxide and IS	Cells treated with peroxide and seeds
Choline	+	0.613	104	12.01±3.78	17.42±0.40	20.23±4.59	13.72±8.68	11.29±3.81
Betaine	+	0.649	118	2.27±0.61	2.17±0.37	2.76±0.50	2.95±1.29	2.45±0.47
Acetylcarnitine	+	0.825	204	0.44±0.35	2.91±2.06	2.51±1.80	ND	0.91±1.28
LPC C16:0	+	0.64	258	0.93±0.07	0.80±0.09	0.81±0.15	0.81±0.31	0.86±0.24
CDP-Choline	+	11.849	489	0.28±0.40	0.30±0.22	0.49±0.37	0.47±0.33	0.39±0.34
Glycerophosphocholine	+	11.757	496	2.43±1.00	3.39±1.22	3.57±2.08	6.53±4.30	5.95±3.36
LCP C18:0	+	10.522	524	1.32±1.16	0.59±0.42	1.55±1.11	1.35±1.3	0.58±0.41
Palmitic Acid	-	11.728	255	0.63±0.20	0.21±0.15	0.18±0.14	ND	0.31±0.11
DCA	-	7.889	391	0.11±0.08	0.15±0.01	0.18±0.10	0.16±0.03	0.18±0.06
Resolvin D1/D2	-	7.096	375	0.6±0.09	0.82±0.06	0.94±0.42	1.13±0.14	1.02±0.02
Arachidonic Acid	-	11.935	303	0.62±0.14	0.35±0.09	0.31±0.17	0.46±0.22	0.57±0.09
LTD4	-	7.137	495	0.27±0.05	0.32±0.05	0.31±0.06	0.27±0.05	0.30±0.03
